# Identification of novel 3D-genome altering and complex structural variants underlying retinitis pigmentosa type 17 through a multistep and high-throughput approach

**DOI:** 10.3389/fgene.2024.1469686

**Published:** 2024-10-23

**Authors:** Suzanne E. de Bruijn, Daan M. Panneman, Nicole Weisschuh, Elizabeth L. Cadena, Erica G. M. Boonen, Lara K. Holtes, Galuh D. N. Astuti, Frans P. M. Cremers, Nico Leijsten, Jordi Corominas, Christian Gilissen, Anna Skowronska, Jessica Woodley, Andrew D. Beggs, Vasileios Toulis, Di Chen, Michael E. Cheetham, Alison J. Hardcastle, Terri L. McLaren, Tina M. Lamey, Jennifer A. Thompson, Fred K. Chen, John N. de Roach, Isabella R. Urwin, Lori S. Sullivan, Susanne Roosing

**Affiliations:** ^1^ Department of Human Genetics, Radboud University Medical Center, Nijmegen, Netherlands; ^2^ Center for Ophthalmology, Institute for Ophthalmic Research, University of Tübingen, Tübingen, Germany; ^3^ Human Genetics Center, School of Public Health, University of Texas Health Science Center, Houston, TX, United States; ^4^ West Midlands Regional Genetics Laboratory, Birmingham Woman’s and Children’s NHS Foundation Trust, Birmingham, United Kingdom; ^5^ Institute of Cancer and Genomic Sciences, University of Birmingham, Edgbaston, United Kingdom; ^6^ UCL Institute of Ophthalmology, University College London, London, United Kingdom; ^7^ Centre for Ophthalmology and Visual Science, The University of Western Australia, Perth, WA, Australia; ^8^ Department of Medical Technology and Physics, Australian Inherited Retinal Disease Registry and DNA Bank, Sir Charles Gairdner Hospital, Perth, WA, Australia; ^9^ Department of Ophthalmology, Royal Perth Hospital, Perth, WA, Australia

**Keywords:** gene diagnostics, gene regulation, inherited retinal dystrophies, retinitis pigmentosa, structural variants

## Abstract

**Introduction:**

Autosomal dominant retinitis pigmentosa type 17 (adRP, type RP17) is caused by complex structural variants (SVs) affecting a locus on chromosome 17 (chr17q22). The SVs disrupt the 3D regulatory landscape by altering the topologically associating domain (TAD) structure of the locus, creating novel TAD structures (neo-TADs) and ectopic enhancer-gene contacts. Currently, screening for RP17-associated SVs is not included in routine diagnostics given the complexity of the variants and a lack of cost-effective detection methods. The aim of this study was to accurately detect novel RP17-SVs by establishing a systematic and efficient workflow.

**Methods:**

Genetically unexplained probands diagnosed with adRP (n = 509) from an international cohort were screened using a smMIPs or genomic qPCR-based approach tailored for the RP17 locus. Suspected copy number changes were validated using high-density SNP-array genotyping, and SV breakpoint characterization was performed by mutation-specific breakpoint PCR, genome sequencing and, if required, optical genome mapping. *In silico* modeling of novel SVs was performed to predict the formation of neo-TADs and whether ectopic contacts between the retinal enhancers and the *GDPD1*-promoter could be formed.

**Results:**

Using this workflow, potential RP17-SVs were detected in eight probands of which seven were confirmed. Two novel SVs were identified that are predicted to cause TAD rearrangement and retinal enhancer-*GDPD1* contact, one from Germany (DE-SV9) and three with the same SV from the United States (US-SV10). Previously reported RP17-SVs were also identified in three Australian probands, one with UK-SV2 and two with SA-SV3.

**Discussion:**

In summary, we describe a validated multi-step pipeline for reliable and efficient RP17-SV discovery and expand the range of disease-associated SVs. Based on these data, RP17-SVs can be considered a frequent cause of adRP which warrants the inclusion of RP17-screening as a standard diagnostic test for this disease.

## Introduction

Retinitis pigmentosa type 17 (RP17, MIM:600852), a form of dominantly inherited retinal dystrophy (IRD), was considered a genetic mystery for several decades (Bardienb et al., 1995; den Hollander et al., 1999). The genetic cause of disease was linked to a region on chr17q22 (hence “RP17”) in the early nineties, but a conclusive genetic cause could not be found. With the arrival of genome sequencing, complex structural variants (SVs) affecting the RP17 locus were discovered as the underlying genomic cause of RP17 (de Bruijn et al., 2020).

To date, eight unique complex RP17-SVs have been described including duplications, duplication-inversions, a triplication and more complex events that affect neighboring genes in the region including *GDPD1* (MIM:616317), *SMG8* (MIM:613175)*,* and *YPEL2* (MIM:609723) (de Bruijn et al., 2020). Causative variants were identified in 22 unrelated families diagnosed with autosomal dominant RP (adRP), encompassing more than 300 affected individuals suggesting this is a major, and until recently unrecognized, locus for adRP. While all currently known RP17-SVs are distinct and have unique breakpoints, the shared feature of all SVs is a rearrangement of the 3D chromosome structure of the RP17 locus. The predicted consequence of the SVs is the creation of novel topologically associating domains (TADs) which was supported by experimental data obtained using RP17-derived retinal organoids. TADs are 3D chromatin domains that exist across the genome and are flanked by boundary elements which restrict chromatin interactions between regulatory sequences such as gene enhancers and promoters (Rao Suhas et al., 2014). Disruption of TAD structures can lead to loss of interaction between enhancers and their target genes or the formation of novel active domains (called neo-TADs) with ectopic contacts occurring between regulatory regions and a new target gene, resulting in pathogenic alterations in gene expression (Spielmann et al., 2018; Franke et al., 2016; Lupiáñez et al., 2015; Ibrahim and Mundlos, 2020). The neo-TADs created by the RP17-SVs facilitate the interaction between active retinal enhancer elements and the promoter of the *GDPD1* gene (de Bruijn et al., 2020). *GDPD1* is only expressed at low levels in healthy photoreceptor cells of the retina, and ectopic expression of *GDPD1* is considered the likely pathogenic mechanism underlying RP17, nevertheless this hypothesis requires further experimental validation.

Based on the number of RP17-affected individuals identified so far, we expect there are numerous RP17-affected individuals who are currently undiagnosed and that RP17 patient numbers will significantly increase if accurate methods are established to efficiently identify these SVs. However, currently the identification of RP17-SVs and the unique breakpoints involved in each SV is challenging. To address this issue, in the current study we have developed a cost-effective strategy to screen for RP17-SVs that could be implemented in standard genetic diagnostic pipelines for IRDs, particularly adRP. Our pipeline is based on a smMIPs- or genomic qPCR-based approach tailored to the RP17 locus in which minimally duplicated regions are included, which enabled accurate detection of known and potentially novel RP17-SVs, all of which are unique. Suspected RP17-SVs were validated by high-density SNP-array genotyping and breakpoints were characterized by mutation-specific breakpoint PCR or genome sequencing. To predict the pathogenicity of the newly identified SVs, hypothetical modeling of the RP17 TAD landscape and inferred consequences of the variants was performed. Both smMIPs-based and qPCR-based approaches were found to be highly efficient for RP17-SV detection. The RP17-SVs we identified included both previously reported SVs (UK-SV2 and SA-SV3) and two, newly identified RP17-SVs which were characterized in detail. New RP17 families were identified within Europe, Australia and the United States, which extends the global prevalence of RP17.

Based on our findings, we advocate that RP17 screening should be part of standard diagnostic genetic screening for adRP and propose a high throughput and cost-effective multi-step workflow. Furthermore, we provide additional evidence and new insights regarding the pathogenic mechanism underlying RP17 as well as a framework for pathogenicity criteria for SVs that affect this locus.

## Materials and methods

### Ethics approval

The study adhered to the tenets of the Declaration of Helsinki and was approved by the local ethics committees of the Radboud University Medical Center (Nijmegen, Netherlands); the University Hospital of Tübingen (349/2003V and 116/2015BO2), the University of Texas Health Science Center at Houston (UTHealth) and the Sir Charles Gairdner Osborne Park Healthcare Group (RGS04985). Written informed consent was obtained from all individual participants before examination, DNA analysis and inclusion in this study.

### Clinical examination

Clinical data were collected from the respective affiliated medical centers for all index cases and thoroughly reviewed, including detailed retinal imaging, fundus autofluorescence, and optical coherence tomography. Information about the inheritance pattern and additional affected family members was obtained through a questionnaire. The clinical status of family members was not re-evaluated in the current study and was based on the index case’s report.

### Genomic qPCR

A quantitative real-time PCR (qPCR) experiment on genomic DNA was designed to allow cost-effective screening for copy number changes affecting the RP17 locus (*GDPD1* – *LINC01476*, chr17:59,220,511–59,526,851). In each qPCR experiment, a known RP17 proband (NL-SV1) was included as positive control. qPCR was performed using the QuantiTect SYBR Green PCR kit (Qiagen, Hilden, Germany) on an Applied Biosystems 7,500 Real Time PCR system. Two pairs of genomic qPCR primers were designed: one pair overlapping with exon 3 and intron 3 of *GDPD1* (genomic region duplicated in seven out of eight known RP17-SVs) and one pair overlapping with intron 2 of *LINC01476* that is enriched with retinal enhancer elements (region involved in all known RP17-SVs) (de Bruijn et al., 2020). Primers located in *RPPH1* (MIM:608513) were used as a reference for standard quantity. Primer sequences and (genomic) positions are listed in Supplementary Table S1.

Each reaction was performed in duplicate and was comprised of 2x QuantiTect SYBR Green PCR Master Mix, 0.6 µL of each primer (10 µM) and 10 ng DNA in a total reaction volume of 20 µL. Cycling conditions were as follows: 95°C for 15 min, followed by 40 cycles at 94°C for 15 s, 60°C for 60 s and 72°C for 37 s. Dissociation melt curves were generated by heat denaturation over a temperature gradient from 69°C to 95°C. Data were analyzed using the 7,500 Software v2.0.6. The fold change of the RP17 target regions was normalized to the wildtype reference genomic region (*RPPH1*), and was calculated using the ΔΔCt method (Pfaffl, 2001).

### smMIPs design and sequencing

For smMIPs-based sequencing, the RP-LCA smMIPs panel was used as previously described (Panneman et al., 2023). The panel includes 417 smMIPs covering the RP17 locus intermittently (chr17:59,220,609–59,430,920 *GDPD1*-*LINC01476*), and accurate detection of known RP17-SVs was previously validated using known RP17 probands (Panneman et al., 2023). Copy number variant (CNV) analysis was performed using an established CNV calling pipeline that was adapted for smMIPs sequencing (Plagnol et al., 2012). In addition, a negative control data set encompassing 100 probands that were genetically explained by homozygous or compound heterozygous pathogenic single nucleotide variants (SNVs) (ACMG/AMP guidelines) was included to improve specificity. All CNVs with a Bayes factor (BF) of ≥100 and an internal frequency of ≤10% in the CNV output were selected for detailed interrogation. The selected BF value cut-off allowed for the robust detection of known RP17-SVs in five RP17-probands that were included in previous sequencing runs using the RP-LCA smMIPs panel containing more than 4,000 patients. All CNVs called in the RP17 locus were subjected to visual inspection using the IGV software (v2.4). If both breakpoints of the CNV could be readily recognized and indicated that the variant is unlikely to cause alteration of the TAD landscape of the RP17 locus considering the size and position of the variant, the variant was excluded from further analysis. Prioritized and suspected alterations in the RP17 region were subsequently validated using high-density SNP genotyping.

To rule out the presence of other pathogenic variants in IRD-associated genes in these probands, SNV analysis was performed following the filtering and prioritization steps described previously (Panneman et al., 2023). In short, SNVs and indels were filtered based on the minor allele frequency (MAF) in gnomAD V2.1.1 (Chen et al., 2024) (MAF ≤0.005 for autosomal recessive IRD genes and MAF ≤0.001 for autosomal dominant IRD genes). Remaining variants were prioritized based on their predicted effect on protein sequence. First, all stop-gain, stop-loss, frameshift, start-loss, and canonical splice site variants were prioritized followed by in-frame insertions and/or deletions. Thereafter, missense variants that were predicted to be disease causing by at least one *in silico* tool (CADD-PHRED (Kircher et al., 2014) score ≥15 [range 0–48] or REVEL (Ioannidis et al., 2016) score ≥0.3 [range 0-1]), or putative splice-altering variants (SpliceAI (Jaganathan et al., 2019) score ≥0.2 [range 0-1]) were prioritized. In addition, a manual CNV analysis was used to rule out the presence of pathogenic CNVs outside the RP17 locus. After filtering and prioritization, ACMG classes were assigned to all variants according to the ACMG/AMP guidelines using the Franklin Genoox Platform (https://franklin.genoox.com/). Based on these classifications, probands were considered possibly or very likely solved based on criteria published previously (Panneman et al., 2023).

### SNP genotyping

SNP genotyping was performed on the CytoScan™ HD array, which contains over 2.6 million probes of which ∼750,000 are SNP-probes (Affymetrix, Inc., Santa Clara, CA, United States). Genomic DNA was hybridized to the array following manufacturer’s instructions. CNVs were called using Affymetrix^®^ Chromosome Analysis Suite (ChAS) with default settings, and data were analyzed using the ChAS software. An overview of the SNP-array data of all currently known RP17-SVs can be found in Supplementary Figure S1. For UK-SV6, the SNP-array data showed some discrepancies compared to the genome sequencing data interpretation in our previous report (de Bruijn et al., 2020). Therefore, this variant was reanalyzed and characterized in detail and the nomenclature and structure was updated accordingly (described in Supplementary Results, Supplementary Figures S2, S3).

### Genome sequencing and variant prioritization

Genome sequencing was performed by BGI (Hongkong, China) as previously described (de Bruijn et al., 2023; Fadaie et al., 2021a). In short, read mapping to the Human Reference Genome build GRCh38/hg38 was performed using Burrows-Wheeler Aligner V.0.7814 (Li and Durbin, 2009). SNV calling was performed using Genome Analysis Toolkit HaplotypeCaller (McKenna et al., 2010), CNV calling using Canvas Copy Number Variant Caller (Roller et al., 2016) (read-depth evidence) and SV calling using Manta Structural Variant Caller V.1.1.0 (Chen et al., 2016) (paired end and split read evidence). Called variants were verified and visualized using the IGV software (V.2.4) (Robinson et al., 2011). SVs and CNVs disrupting the RP17 locus were interrogated in detail and assessed for putative pathogenicity by TAD remodeling.

For each affected individual in which a novel candidate RP17-SV was identified, an additional genome-wide SNV and CNV/SV analysis was performed to exclude the presence of other potentially pathogenic variants. All variants overlapping with adRP-associated genes (RetNet, https://sph.uth.edu/retnet/, accessed first of March 2024) were selected and analyzed. Variants were prioritized based on a MAF ≤0.001 in gnomAD v.3.1 (Chen et al., 2024) (SNVs) or 1000G (Fairley et al., 2020) (SVs and CNVs). For SNV prioritization: stop-loss or -gain, start-loss or gain, frameshift, in-frame deletion or insertion variants were selected. Missense variants and candidate splice variants were evaluated following abovementioned criteria based on CADD-PHRED (Kircher et al., 2014), REVEL (Ioannidis et al., 2016) and SpliceAI (Jaganathan et al., 2019) predictions. SVs and CNVs were prioritized when disrupting the coding region of a candidate gene.

### Breakpoint PCR and Sanger sequencing

Suspected SV breakpoint junctions derived from SNP-array data (known SVs, Supplementary Figure S1 NL-SV1 – UK-SV8) or genome sequencing data (novel SVs) were PCR-amplified and validated with Sanger sequencing. Primer sequences and coordinates of all RP17-SVs are listed in Supplementary Table S2, and PCR conditions are available upon request.

## Results

### Experimental design of RP17 screening methods

**FIGURE 1:**
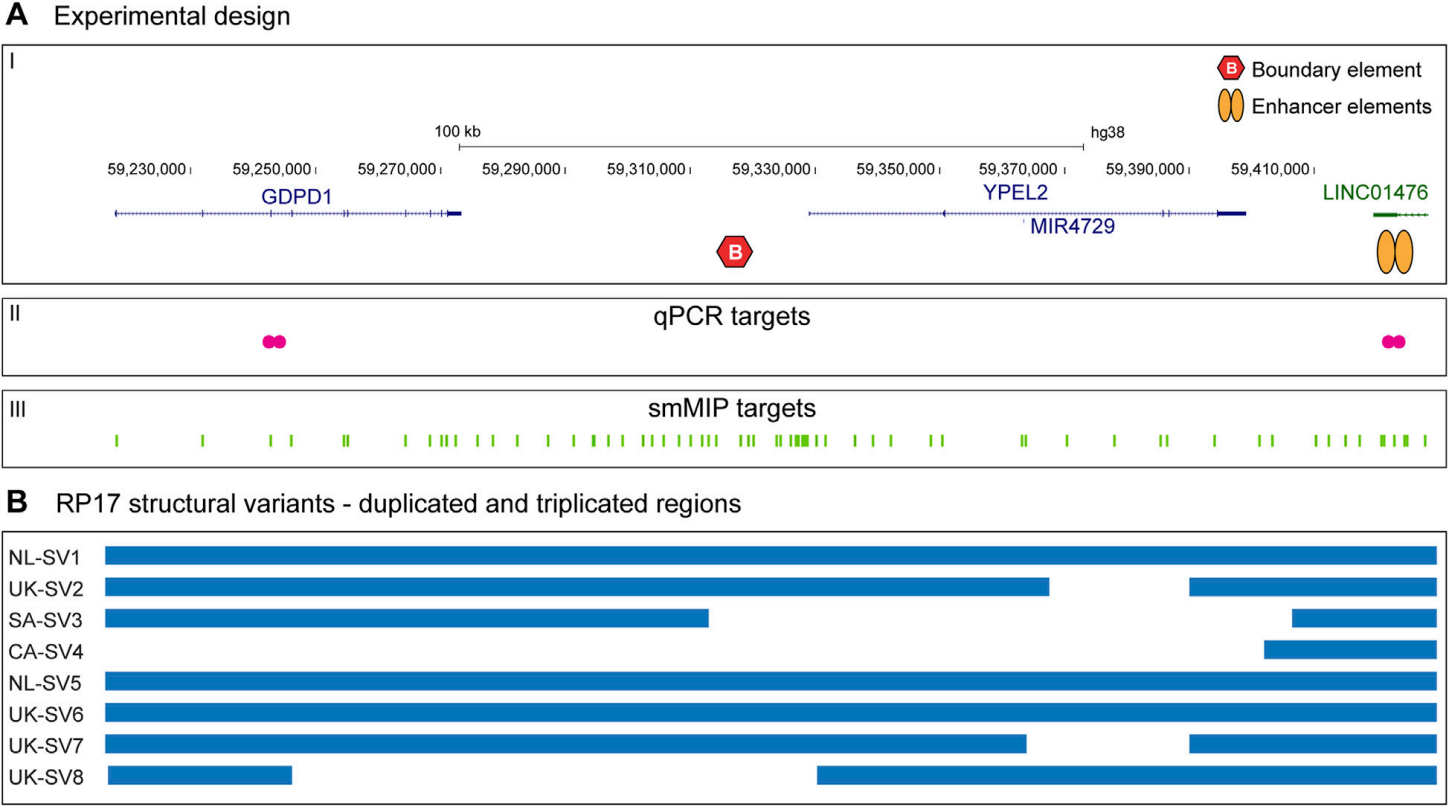
Schematic overview of the study design and RP17 screening targets. **(A-I)** The top panel provides a schematic overview of the RP17 locus including the *GDPD1*, *YPEL2* and *LINC01476* genes, a CTCF-enriched boundary element and active retinal enhancer elements that are located in *LINC01476*. **(A-II)** To allow efficient screening of copy gains overlapping with the RP17 locus, two sets of genomic qPCR probes were designed that span the exon 3 - intron 3 boundary of *GDPD1* and located in intron 2 of *LINC01476*. **(A-III)** In addition, 417 smMIPs probes were designed that are distributed over the complete RP17 locus. *LINC01476* is only partially displayed in the figure. **(B)** Overview of previously identified RP17-associated structural variants and duplicated and triplicated genomic regions involved.

RP17-SVs are characterized by duplications and unique breakpoints in specific regions of the RP17 locus (chr17q22). Based on all previously identified pathogenic RP17-SVs (de Bruijn et al., 2020), we determined a minimally implicated region from *GDPD1* to *LINC01476* that is involved in copy number changes (duplications and/or triplications) as well as more complex duplication-inversion events. We explored the use of both genomic qPCR- and smMIPs-based screening to efficiently detect copy number changes affecting this critical region in the RP17 locus (Figure 1).

### Genomic qPCR reveals novel German RP17 structural variant (DE-SV9)

**FIGURE 2:**
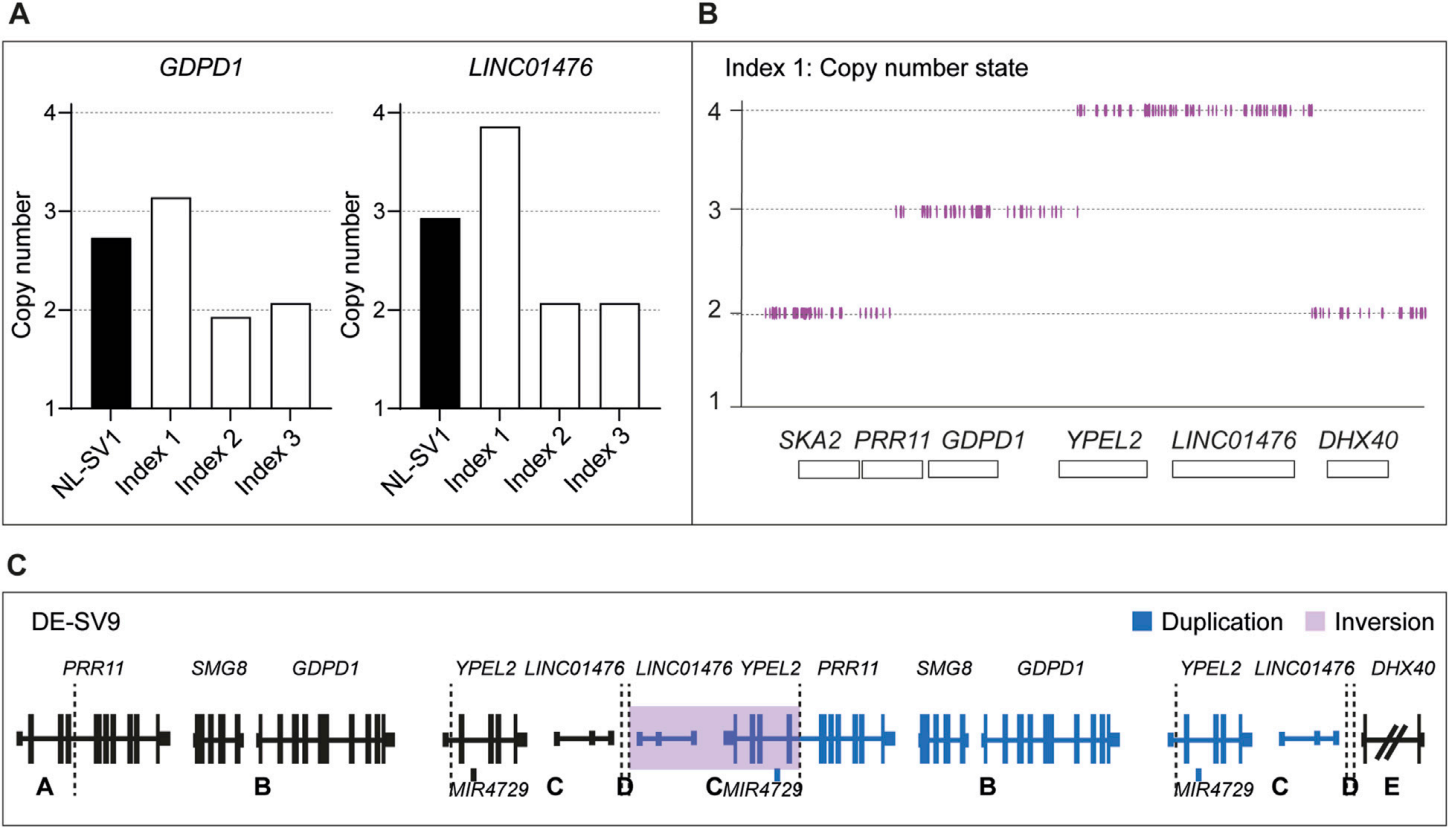
Genomic qPCR detects a novel RP17-SV in German index patient. **(A)** In total 230 individuals affected with dominant retinitis pigmentosa were screened for copy number changes in the RP17 region by genomic qPCR. Genomic DNA from an individual carrying NL-SV1 was included as a positive control. In one index case of German origin (Index 1), genomic qPCR suggested a possible duplication of *GDPD1,* and a triplication of *LINC01476* that is enriched for retinal enhancer elements. Index 2 and Index 3 were included in the figure as two representative negative samples that were screened by genomic qPCR. **(B)** qPCR results of index 1 were confirmed by SNP-array genotyping. **(C)** Since the identified structural variant (SV) did not resemble any of the previously reported RP17-SVs, genome sequencing was performed to determine the breakpoints of the variant. Genome sequencing confirmed the presence of a complex SV, which was termed DE-SV9. Breakpoints are indicated with dashed lines. Blue segments represent duplicated (B, D) or triplicated (C) regions. Inversions are highlighted in purple (segment C). The size of *DHX40* is reduced for the purpose of the figure.

A German cohort of 230 adRP-affected individuals was subjected to genomic qPCR-based screening. The affected individuals received a varying degree of prescreening ranging from no prescreening, to *RHO*-targeted sequencing, gene panel-based sequencing and exome sequencing. For one affected individual, qPCR results indicated a potential duplication and/or triplication event within the RP17 locus (Figure 2A). To confirm the suspected SV, SNP genotyping on a high-density SNP-array was performed. Results confirmed the presence of an RP17-SV and suggested a novel complex SV that included both a duplication and a triplication event (Figure 2B). Since the candidate SV did not resemble any of the previously identified RP17-SVs (Supplementary Figures S1, S4), genome sequencing was performed to determine the exact breakpoints of the SV. The complex SV was efficiently detected by both CNV and SV calling, and included duplication, triplication and inversion calls (Figure 2C; Supplementary Table S3). Breakpoints of the complex SV (termed DE-SV9) were confirmed by Sanger sequencing. Segregation analysis by mutation-specific breakpoint PCR (data not shown) confirmed cosegregation of the SV with the adRP phenotype in the family (Supplementary Figure S5). Genome sequencing analysis revealed no other potentially pathogenic variants affecting adRP-associated genes.

### smMIPs screening reveals several candidate RP17 structural variants

**FIGURE 3:**
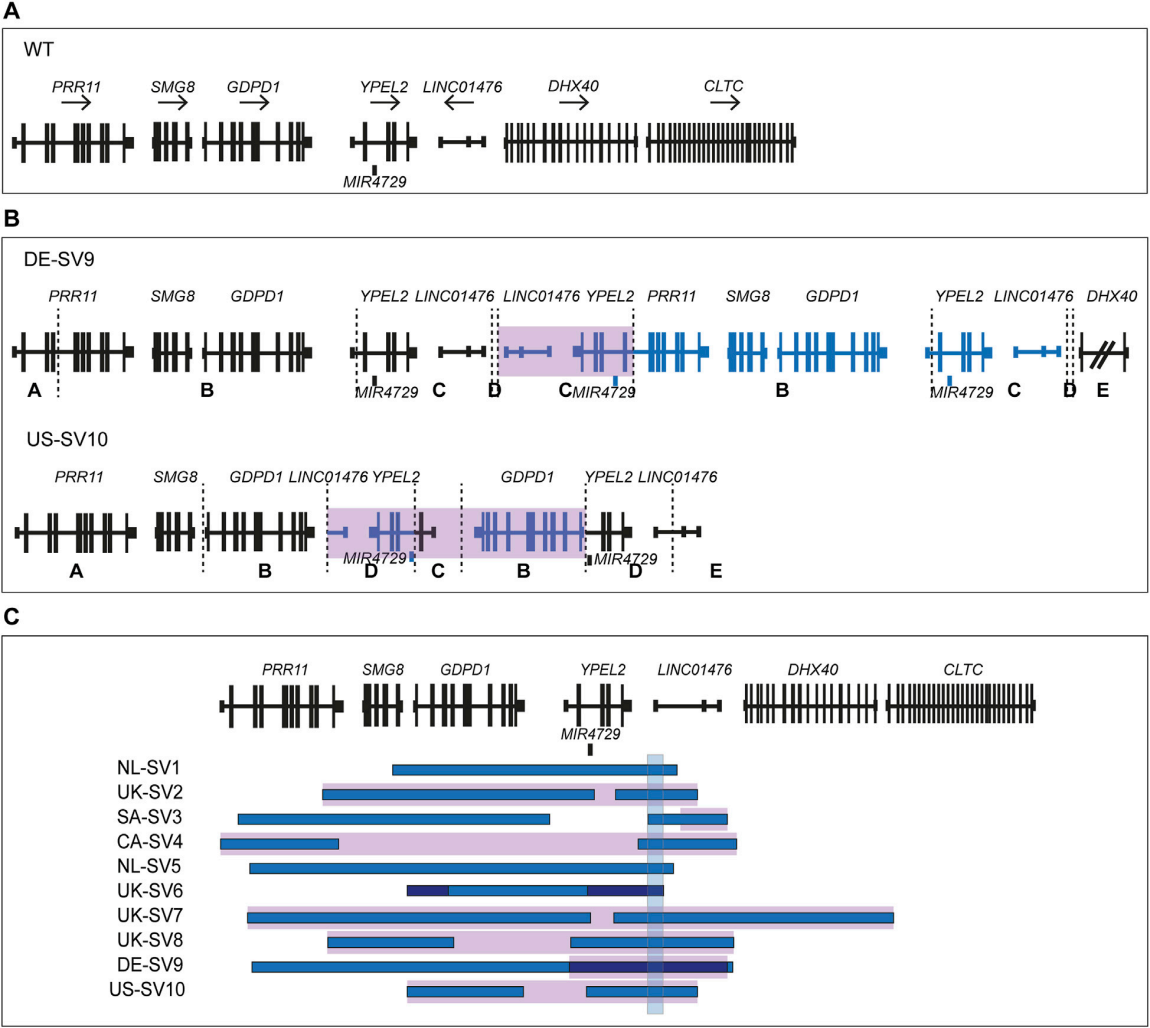
Overview of novel structural variants within the RP17 locus in adRP families. Breakpoints are indicated with dashed lines. Blue segments represent duplicated or triplicated regions, whereas inversions are highlighted in purple. **(A)** Wildtype (WT) chromosomal localization of the RP17 locus. **(B)** Novel RP17 structural variants identified in a German adRP family (DE-SV9) and a large US adRP family (US-SV10). **(C)** Overview of all SV breakpoints identified in the RP17 locus. An overlapping genomic region that is duplicated or triplicated in all reported pathogenic RP17-SVs is highlighted with a blue box (de Bruijn et al., 2020). This region was found to be duplicated or triplicated in all the newly identified SVs (chr17:59,421,853–59,433,404, 11.5 kb). Based on recent data acquired using this approach (Supplementary Results), the structure and nomenclature of UK-SV6 has been revised from the previous report (de Bruijn et al., 2020).

smMIPs-based sequencing of the RP17 locus was performed for 279 adRP probands primarily originating from Australia and the United States. In seven of the adRP probands, a CNV overlapping with the RP17 region was detected. High-density SNP genotyping was performed to confirm the presence of the SV and enable comparison to the previously identified and published RP17-SVs. For one of the probands, no indication of copy number changes in the SNP-array results was detected and this variant call was deemed false positive (BF-value CNV call of 206). In the remaining six samples, a variety of copy number changes were observed. Three of the samples revealed copy number changes that were comparable to previously reported SVs (Supplementary Figures S6A, S6B). This was confirmed by PCR amplification of mutation-specific breakpoints (UK-SV2, n = 1 and SA-SV3, n = 2). The other three samples, all originating from the United States, showed a similar copy gain pattern that did not resemble any of the known RP17-SVs (Supplementary Figure S6C). A more detailed interrogation of the family history revealed a distant relationship between these individuals (Supplementary Figure S5). To determine the breakpoints of the suspected novel SV, genome sequencing was performed for one individual. Guided by the genome sequencing results, a novel RP17-SV, labelled US-SV10, was further confirmed by PCR-based breakpoint validation (data not shown) and segregation analysis (Supplementary Table S3; Figure 3; Supplementary Figure S5). No other candidate variants affecting adRP-associated genes were identified by genome sequencing analysis.

### Modeling of novel RP17-SVs to investigate possible *YPEL2* TAD-disruption

To assess the potential pathogenicity of the newly identified RP17-SVs, DE-SV9 and US-SV10, *in silico* modeling of the TAD landscape was performed. The epigenetic wildtype landscape of the RP17 locus, including TADs, TAD boundaries, active retinal enhancer elements and their approximate genomic coordinates were retrieved from existing epigenetic data resources (Cherry et al., 2020) as previously described (Supplementary Figure S7) (de Bruijn et al., 2020). Hypothetical modeling of the TAD structure for the novel SVs shows that, similar to the other RP17-SVs, *GDPD1* enters a neo-TAD creating an ectopic contact with the retinal enhancer elements (Supplementary Figure S8). These findings are in line with the previously suggested disease mechanism underlying RP17. Although the ACMG/AMP variant classification guidelines (Richards et al., 2015) are mainly designed for the classification of protein coding variants, we attempted to classify the novel identified variants following the guidelines described by (Ellingford et al., 2022). Based on these recommendations, DE-SV9 was classified as likely pathogenic (PM2, PM5, PP1, PP3) and US-SV10 as pathogenic (PP1_strong (Jarvik and Browning, 2016), PM2, PM5, PP3). We consider both these variants to be the likely genetic cause of the adRP within these families.

### Assembly of an RP17 structural variant database

To provide a complete catalogue of reported RP17-SVs, all variants were uploaded into the Leiden Open Variation Database (www.lovd.nl/gdpd1, www.lovd.nl/smg8 and www.lovd.nl/ypel2) and all findings can be accessed via https://databases.lovd.nl/shared/diseases/02318. Nomenclature of the RP17-SVs was updated and corrected according to the most up to date HGVS-guidelines v20.05 (den Dunnen et al., 2016) (adjustments were implemented to the previously reported nomenclature in de Bruijn et al., 2020 (de Bruijn et al., 2020)). The LOVD database now provides a complete description of all RP17-SVs including their pathogenicity, which will be updated and actively curated. An overview of the genomic details, origin and prevalence of all previously reported and newly identified RP17-SVs is shown in Table 1.

**TABLE 1:** Overview of RP17 structural variants and affected individuals.

SV	HGVS nomenclature	Origin	# Affected individuals
NL-SV1	g.59214554_59440776dup	The Netherlands	37^3^ (1 family)
UK-SV2	g.59378750_59391598delins[59198478_59481750inv;AGGCTGGTC]	United Kingdom	123^3,10,*^ (14 families)
SA-SV3	g.59439317_59439318ins[AAAAAAAACTTGAAAAAGAAGTTTG 59170259_59314314; 59439322_59535354inv; GGTCCAGATTGTG;59421853_59439317]	South Africa	120^3,*^ (6 families)
CA-SV4	g.59202647_59406522delins[59155674_59557539inv;TAAGCA]	Canada	42^3^ (1 family)
NL-SV5	g.59183160_59438501dup	The Netherlands	11^3^ (1 family)
UK-SV6	g.59247345_59247346ins[A;59362745_59433393; 59218621_59433393; 59218621_59247345]	United Kingdom	25^3^ (1 family)
UK-SV7	g.59376270_59391569delins[59182164_59633460inv;TT]	United Kingdom	2^3^ (1 family)
UK-SV8	g.59248874_59335791delins[CT;59199986_59554298inv]	United Kingdom	12^3^ (1 family)
DE-SV9	g.59549138_59549139ins[59336282_59545765inv;59187321_59545765]	Germany	4^*^ (1 family)
US-SV10	g.59288296_59362561delins[59220112_59478159inv;A]	United States	16^*^ (1 family)

Overview of all reported structural variants affecting the RP17 locus on chr17q22. Genomic coordinates are according the GRCh38/hg38 genome assembly and variant nomenclature is established following the HGVS, guidelines. All variants have been uploaded to the Leiden Open Variation Database (see LOVD.nl/GDPD1, LOVD.nl/SMG8 and LOVD.nl/YPEL2). Based on recent data acquired using our approach (Supplementary Results), the structure and nomenclature of UK-SV6, has been revised compared to the previous report (de Bruijn et al., 2020). *, identified in the current study.

## Discussion

**FIGURE 4:**
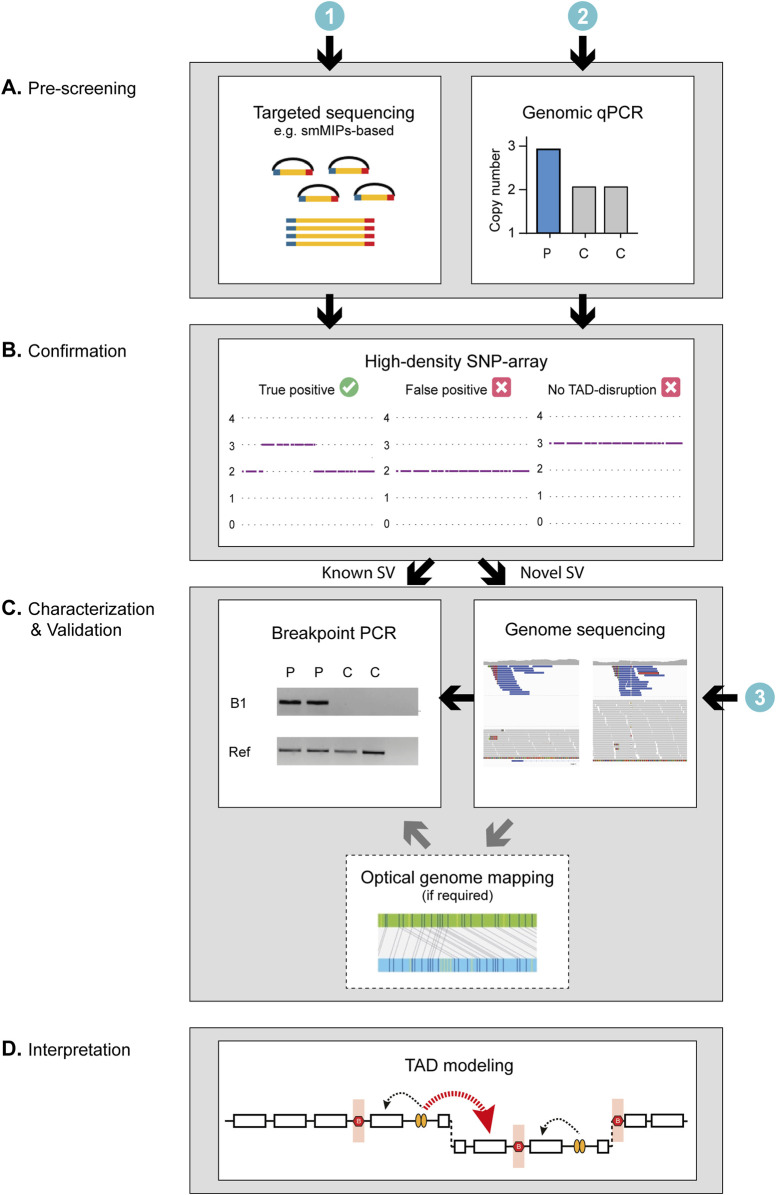
Schematic of the proposed workflow for the screening and characterization of RP17-SVs. The genetic investigation of RP17-SVs can be divided into three steps. **(A)** As a first step, prescreening to identify copy number changes can be performed using (Bardienb et al., 1995) genomic qPCR for specific targets in the RP17 locus or alternatively by (den Hollander et al., 1999) targeted sequencing such as smMIPs-based sequencing with probes designed to cover critical regions of the RP17 locus or exome sequencing. **(B)** Positive cases should be validated by high-density SNP array to discriminate between false- and true-positive variants. For diagnostic facilities that have already implemented (de Bruijn et al., 2020) genome-sequencing as a standard diagnostic test, prescreening steps I and II can be skipped. **(C)** If the SNP array data or variant calls from the genome sequencing data correspond to that of a known SV, a mutation-specific breakpoint (B1) PCR should be performed to confirm the SV identity. If the data suggest a novel SV, the breakpoints need to be determined and genome sequencing (if not performed yet) should be undertaken to characterize the SV in more detail. If the orientation of the SV cannot be resolved using short-read sequencing data only (e.g., as observed for UK-SV6, Supplementary Results) optical genome mapping or long-read sequencing should be performed to fully characterize the novel variant. **(D)** Finally, to assess pathogenicity of RP17-SVs the predicted effect the SV has on the TAD landscape of the RP17 locus should be assessed. For a RP17-SV to be pathogenic, the convergent feature is that there has to be a disruption of boundary elements to allow for ectopic contact between the retinal enhancers and the promoter of *GDPD1* (Supplementary Figure S7). C, control; P, patient; Ref, Reference gene.

RP17 is a form of dominantly inherited retinitis pigmentosa caused by pathogenic structural abnormalities affecting the chr17q22 region. The genetic cause of RP17 was only recently discovered, yet already over 300 affected individuals have been genetically diagnosed with this disease. The uniqueness of RP17-SVs, specifically the different mutational breakpoints and the regions which are duplicated, triplicated and/or inverted, prohibits the use of one universal genetic test to screen for RP17 variants. Thus far, all RP17-SVs were initially characterized and identified using genome sequencing but considering the relative high costs of this technique, a global implementation of this technique is not considered realistic. Given the complexity of previously characterized RP17-SVs, several important factors need to be considered when designing a more cost-effective workflow for RP17-SV detection. Namely, (i) how to confidently characterize the type of variants identified and discriminate tandem duplications from more complex events such as duplicated-inversions, (ii) how to discern false-positive variant calls from true-positive variant calls and finally, (iii) how to predict the effect of the SV on the TAD landscape of the locus, the creation of ectopic enhancer-gene contacts and therefore the pathogenicity of the variant. In this study, we have incorporated these considerations and designed a workflow for rapid and efficient RP17-screening (Figure 4). The suggested workflow consists of three consecutive steps: (Bardienb et al., 1995): Low cost and high-throughput pre-screening, (den Hollander et al., 1999) Validation of suspected copy number changes to discriminate between false- and true-positive variants, (de Bruijn et al., 2020) Confirmation and interpretation of the variant by breakpoint-characterization. In addition, for newly identified RP17-SVs, TAD landscape modeling should be performed to predict the pathogenicity of the variant (Supplementary Figure S7).

We have compared the use of two different low cost pre-screening technologies to indicate whether a proband could potentially harbor a copy number change affecting the RP17 locus. In total, 509 individuals affected with dominant RP were screened, 230 by genomic qPCR approach and 279 by smMIPs-based sequencing. These methods were selected considering the relatively low cost, the requirement of only low quantities of DNA and successful screening of degraded DNA samples (Khan et al., 2020). Both methods successfully detected potential RP17-SVs (1/230 by qPCR, and 7/279 by smMIPs). The accessibility and rapid implementation of the genomic qPCR screening approach could be considered an advantage of this technique over smMIPs-based sequencing. Nevertheless, since the regions targeted by the qPCR-probes is rather limited (2 PCR-amplicons were used in this study) compared to the smMIPs probes (417 probes covering the complete RP17 locus), the sensitivity of this technique could be a potential limitation. Although the regions that are most prone to copy number changes based on previously identified SVs were targeted, we cannot exclude the possibility that novel pathogenic SVs, involving alternative duplicated regions, would be missed. In addition to the German adRP cohort screened in this study, Belgian and Spanish adRP cohorts (145 and 22 affected individuals, respectively (personal communication Prof. De Baere and Dr. Ayuso)) were also screened by genomic qPCR, but no suspected RP17 alterations were found. Known RP17-probands were included as positive controls in both screenings. This could suggest that either no RP17-SVs are present in these (relatively small) adRP cohorts, or that the sensitivity and resolution of the current qPCR-method is too low and that additional qPCR targets should be incorporated to cover more of the RP17-region. To discern between these two hypotheses, it would be valuable to screen these cohorts by smMIPs-based sequencing for an objective comparison between the two approaches.

Based on our experience, we recommend using high-density SNP-based genotyping to validate suspected RP17-changes derived from qPCR- or smMIPs-based data. The use of SNP-genotyping has several advantages. Firstly, the SNP-array allows for the discrimination of false-positive and true-positive variants calls. In this study, we identified one proband with a suspected RP17-SV based on smMIPs-based CNV calling, but no copy number changes were revealed by the SNP-array. We excluded this sample from further analysis and avoided costly follow-up studies. Secondly, in contrast to the pre-screening technologies, SNP-array genotyping does provide an initial size indication of the SV detected. In a previous study described by (Panneman et al., 2023), a large intrachromosomal duplication spanning from q21.32 to q24.2 on chromosome 17 was identified by smMIPs-based sequencing. Only by performing SNP-array genotyping the large size of the duplicated region could be recognized. As no breakpoints were present within the RP17 locus to cause disruption of the RP17-TAD landscape, the variant was considered (likely) benign and was excluded as disease-causing. In addition, multiple, shorter CNV calls distributed over the RP17 locus can be present within one sample which also hampers an accurate size estimation. This limitation of smMIPs-based CNV calling further underlines the importance of SNP-genotyping to estimate the size of the CNV. A third advantage of SNP-array is the ability to recognize previously characterized RP17-SVs based on a one-to-one comparison and the unique patterns of RP17 copy number changes. When a known RP17-SV is suspected, breakpoint-validation by PCR can readily be performed to confirm the presence of the presumed variant. In three out of eight probands we screened by SNP-array in this study, we could confirm the presence of a previously described RP17-SV (i.e., UK-SV2 and SA-SV3). Nevertheless, based on our results, we have observed that the resolution of the SNP-array data is limited and the technique lacks base-pair-resolution. It is important to keep in mind that the called breakpoints based on the SNP-array should be considered “rough estimates” because of the resolution of the technique. As a consequence, called breakpoints can slightly differ between individuals which hampers the recognition of known RP17-SVs (Supplementary Figure S6). If SNP-genotyping suggested a potential novel RP17-variant, we performed genome sequencing to characterize the variant. By combining the use of SV- (split-read evidence) and CNV- (copy number changes) callers, the SV type and for the majority of SVs, the orientation could be determined.

For diagnostic facilities that have implemented genome-sequencing as a standard diagnostic test, the pre-screening steps (Figure 4A) can potentially be skipped. However, based on our results, the importance of using the most recent SV- and CNV-caller pipelines should be emphasized. Previously, UK-SV6 was described as a full triplication event, however, our recently generated SNP-array data suggested a duplication and only a partial triplication event (Supplementary Figure S1; Supplementary Results). Based on these findings we reanalyzed the previously generated genome sequencing data using our updated SV-pipeline (combining SV- and CNV-callers (Manta (Chen et al., 2016) and CANVAS (Roller et al., 2016), respectively). Re-analysis revealed an additional mutational breakpoint indicating a deletion and the partial triplication was confirmed. Since the orientation of the SV could not be resolved completely based on the short-read data, optical genome mapping was performed. This highlights that for some SVs, long-read approaches might be required to fully resolve the structure of an SV and hence its pathogenicity. Additionally, it is possible that true RP17-SVs can still be overlooked using only short-read sequencing approaches. There are various reports of pathogenic, complex SVs that were only identified after employing long-read approaches such as long-read genome sequencing (e.g., SMRT- or ONT-sequencing) or optical genome mapping (Fadaie et al., 2021b; Schrauwen et al., 2024). Based on our results, we have adapted the nomenclature and structure of UK-SV6, including the report in the LOVD database.

The proposed pathogenic mechanism underlying RP17 is the creation of neo-TADs which allow ectopic enhancer-gene contacts between retinal enhancer elements and the *GDPD1* promoter. Since not all SVs affecting the RP17 locus allow the creation of neo-TADs and *GDPD1*-enhancer contact, and would therefore be likely benign, it is extremely important to model the putative effect of each SV on the TAD landscape of the locus. Based on existing epigenetic datasets, we have provided the approximate coordinates of the retinal enhancer elements, and CTCF boundaries (Supplementary Figure S7). Previously, alteration of the 3D chromatin landscape of one RP17-SV (UK-SV2) was determined by a low-C method (de Bruijn et al., 2020; Díaz et al., 2018). However, for the remaining RP17-SVs, the hypothetical modeling is still considered a prediction of how the TAD landscape is rearranged. Functional characterization of the variants and the epigenetic hallmarks will be required to understand why these SVs are pathogenic and the convergent mechanism of disease.

The number of RP17-variants identified so far suggests this region is prone to chromosomal aberrations. The proposed RP17-disease mechanism, which involves ectopic gene expression, is considered a rare disease mechanism, which needs to be investigated in more detail to better understand whether this could be a phenomenon in other rare (Mendelian) diseases. In addition to the RP17 locus, other loci have been described that are prone to structural abnormalities and multiple pathogenic SVs have been reported. For example, different disruptions of the *EPHA4* TAD structure have been associated with limb malformations. In contrast to the RP17 locus, different types of SVs affecting the *EPHA4* locus results in different phenotypic outcomes i.e., deletions are associated with brachydactyly (short fingers) whereas duplications are associated with polydactyly (additional fingers) (Lupiáñez et al., 2015). An oncogene-related study also indicated that TAD-boundary deletions can be pathogenic (Hnisz et al., 2016). This suggests that deletion of boundary elements can also lead to gene mis-regulation, as well as other types of SVs, such as copy-neutral variants (inversions or translocations). Copy-neutral variants would, however, be missed by our proposed pre-screening methods and could be considered a limitation of the proposed experimental paradigm.

In conclusion, by following the proposed workflow, we were able to establish a genetic diagnosis for seven affected probands who are part of five larger adRP families that contain 46 affected individuals. Taken together with previous findings, the number of RP17-affected individuals rises to 392 and include individuals from South Africa, Australia, North America and Europe. Among the identified SVs are two novel RP17-SVs (DE-SV9 and US-SV10), which suggests that the RP17 locus is a mutational hotspot for adRP, and that more novel RP17-SVs may be identified by effective screening pipelines, such as the one described here. These findings further support our hypothesis that RP17 could be one of the most important contributors to the missing heritability that has been described for adRP and suggests that RP17-screening should be part of routine genetic testing for adRP.

## Data Availability

The data analyzed in this study is subject to the following licenses/restrictions: smMIPs-based and genome sequencing data are subject to controlled access because they may compromise the privacy of research participants. These data may become available upon a data transfer agreement approved by the local ethics committee and can be obtained after contacting the corresponding author. Requests to access these datasets should be directed to Suzanne de Bruijn, Suzanne.deBruijn@radboudumc.nl.
