# Supplementary material for: Identification of novel 3D-genome altering and complex structural variants underlying retinitis pigmentosa type 17 through a multistep and high-throughput approach
Source: Front Genet. 2024 Oct 23;15:1469686. doi: 10.3389/fgene.2024.1469686 (PMC11537883; doi:10.3389/fgene.2024.1469686)
Supplement: Supplementary file 1 [file DataSheet1.PDF]

SUPPLEMENTARY INFORMATION

Identification of novel 3D-genome altering and complex structural variants underlying retinitis pigmentosa type 17 through a multistep and high-throughput approach

Suzanne E. de Bruijn, Daan M. Panneman, Nicole Weisschuh, Elizabeth L. Cadena, Erica G.M. Boonen, Lara K. Holtes, Galuh D.N. Astuti, Frans P.M. Cremers, Nico Leijsten, Jordi Corominas, Christian Gilissen, Anna Skowronska, Jessica Woodley, Andrew D. Beggs, Vasileios Toulis, Di Chen, Michael E. Cheetham, Alison J. Hardcastle, Terri L. McLaren, Tina M. Lamey, Jennifer A. Thompson, Fred K. Chen, John N. De Roach, Isabella R. Urwin, Lori S. Sullivan, Susanne Roosing

SUPPLEMENTARY RESULTS

SNP-array data suggest incomplete triplication event for UK-SV6

To create a reference RP17-SNP-array dataset for all previously reported RP17-SVs (1), SNP-genotyping was performed on a high-density SNP-array. UK-SV6 was previously reported (1) to be a full triplication event (triplication of segment "X"), however the SNP-array data suggested a duplication-triplication event instead, including an additional mutation-specific breakpoint (**Supplementary Figure 1**). Previously, the triplication event was validated by genomic qPCR event, however, since the qPCR primers used were located in the triplicated segments and not in the duplicated segments, this event was not detected. To investigate in more detail, we reanalyzed the preexisting genome sequencing data of the index patient harboring UK-SV6 using our most recent SV pipeline (described in **Materials and Methods**) and examined the CRAM-file in IGV (2). This analysis revealed a deletion within one of the presumed triplicated segments (**Supplementary Figure 2**) and confirmed the SNP array findings. New mutation-specific breakpoint primers were designed for the additional deletion breakpoint (**Supplementary Table 2**), and the presence of this breakpoint was confirmed by PCR in the index case.

Optical genome mapping was successfully employed to resolve the orientation of UK-SV6

Based on the short-read genome sequencing data it was not possible to fully resolve the orientation of the adapted UK-SV6, i.e., which one of the triplicated segments (segment "X") contains the deletion. Therefore, to fully resolve the orientation and structure of UK-SV6, optical genome mapping (OGM) was performed. Optical genome mapping (Bionano Genomics, San Diego, CA, USA) was performed as previously described (3). In brief, ultra-high molecular weight DNA was isolated from whole peripheral blood (EDTA) using the SP Blood & Cell Culture DNA Isolation Kit (Bionano Genomics, San Diego, CA, USA). DNA labeling was performed using the DLS (Direct Label and Stain) DNA Labeling Kit (Bionano Genomics, San Diego, CA, USA) and the labeled sample was loaded on a 3×1300 Gb Saphyr chip (G2.3) on a Saphyr instrument (Bionano Genomics, San Diego, CA, USA). Annotated *de novo* assembly was

performed using Bionano Access version 1.7 and Bionano Solve version 3.7, which includes two separate algorithms for SV and CNV detection as described previously.[13]

Based on the OGM data, a *de novo* consensus optical map could be built for the affected UK-SV6 individual and aligned to the hg38 genome build. When interrogating the RP17 locus (chr17q22), the structure of UK-SV6 could be resolved (**Supplementary Figure 3**). The data revealed a deletion (coined "segment X-II)" to be present in the first copy of the involved segment ("X"). Hypothetical modeling of the TAD structure for updated SV was performed, and similar as to previously reported for the originally described UK-SV6, suggest that two copies of GDPD1 enter neo-TAD structures with ectopic contacts with active retinal enhancer elements (**Supplementary Figure 3**). Based on these findings, UK-SV6 is still classified as (likely) pathogenic.

SUPPLEMENTARY TABLES

Supplementary Table 1: Genomic qPCR primers

Target	Genomic region	Primer	Oligonucleotides (5'-3')
GDPD1 exon 3-intron 3	chr17:59,245,415-59,245,564	Forward	GCGGTTAAAATCGGAACTGA
		Reverse	TGCATTTTTACTTACACAGTATTTGA
LINC01476 intron 2	chr17:59,430,726-59,430,825	Forward	TCGTGCCTCTTAAGAACTGG
		Reverse	CTCCTGACTTCGTGATCTGC
RPPH1 exon 1	chr14:20,343,119-20,343,253	Forward	CGCGCGAGGTCAGACT
(reference gene)		Reverse	GGTACCTCACCTCAGCCATT

Supplementary Table 2: Primer sequences utilized to validate and characterize mutation-specific breakpoints

SV	Breakpoint	Coordinates	Forward primer (5'-3')	Reverse primer (5'-3')	Amplicon size (bp)
			**Previously reported structural variants (de Bruijn et al., 2020)**		
**NL-SV1**	B1	59,440,776-59,214,554	GGCACTAATGAAACCAGAAAGACACTTGGC	CCACGGAGCACCTTGTAGCTCATTAACTGC	915
**UK-SV2**	B1	59,378,749-[59,481,750]	ATCAGGCAACACGACACCAT	AGAGTGTTAACAAAGTAGACTCGAT	1262
	B2	[59,198,478]-59,391,599	CCTATCCAGTAAATGCCTCTTCC	AATCCAACACATCTTCAGGGCA	1370
**SA-SV3**	B1	59,439,317-59,170,259	GGGTGTGGTTTGTTTGGTTG	CAAGAGAACAATGCAGTGCG	986
	B2	59,314,314-[59,535,354]	CAGGTGTGTAAAAGTGGCATG	TCCTAGTTATGTTGGGAAGTGC	969
	B3	[59,439,322]- 59,421,853	GAATTTGCTTGAAGGGCTTG	AAGCCAAGATCATCCAAACC	481
**CA-SV4**	B1	59,202,646-[59,557,539]	ATACAGGGAGACCCCGTTTC	CTGATCGAAGTGCAAAATGG	1802
	B2	[59,155,674]- 59,406,523	AACAGGCCCAGCTACTCAAG	CAGCAGCAGCATTATCAACC	492
**NL-SV5**	B1	59,438,501-59,183,160	TTGCACCGCTGTTAAGAAAG	GAAGAGGAGACCCCAAAATG	648
**UK-SV6**	B1*	59,247,345-59,362,745	TGTATTCTCACTGTGCATCATG	CAGTGGATTCTAGTCAGCCC	1219
	B2*	59,433,393-59,218,621	ATGCCCAGAGACCTCAAATC	ACCTGATGACTATGGAAGCG	461
**UK-SV7**	B1	59,376,269-[59,633,460]	ACCTTCATGACCCCATCTTC	CTGTAACTCCTCATGCTGATTG	475
	B2	[59,182,164]-59,391,570	TGAGGAGGGTGTATGGGAAG	ATCTTGGCACCACACAACAC	581
**UK-SV8**	B1	59,248,873-[59,554,298]	TGGGGATGTTCTGCTAAGGG	TGCCTGTAGTCCAATTTCTCAG	458
	B2	[59,199,986]-59,335,792	CCAATGCAAAACCTGATACAGT	CACCAAGCATTTTCAGCAGC	569
			**Novel RP17 structural variants**		
**DE-SV9**	B1	59,549,138-[59,545,765]	TGTAACATCCTCCTCCAAGG	GAAAAGAGAACCGGGTGTG	227
	B2	[59,336,282]-59,187,321	TCCAAAGTCCAGGCTCTTAG	GGATTACAGGCATGCACTAAC	315
**US-SV10**	B1	59,288,295-[59,478,159]	TGACTCAGATGAGGAAAGCTG	ACTATCCAGGTGCGGTAGTG	560
	B2	[59,220,112]-59,362,562	CTGAGCGTTTGTCACCTAGC	GCTAGCAATGGTGCAGATTT	544

Primer sequences for mutation-specific breakpoint testing of SA-SV3, UK-SV6 and UK-SV7 were redesigned and optimized after the previous publication (1). Additionally, nomenclature for mutation-specific breakpoints of UK-SV6 have been revised as described in the **Supplementary results**. SV, Structural variant; Breakpoint, Mutation-specific breakpoint (B); Coordinates, Genomic positions of mutation-specific breakpoints according to GRCh38/hg38, [] indicates inverted segment.; Forward primer and reverse primer, Primer sequences used for PCR amplification and Sanger sequencing; Amplicon size, Size of amplified PCR product in base pairs (bp).

Supplementary Table 3: Genomic details of novel RP17-SVs

SV	Type	Chr	Start	End	Event	Size (Mb)	Genomic regions	Genes involved
**DE-SV9**	Complex	17	59,187,321	59,549,138	Dup	0.36	B, C, D	*PRR11, SMG8, GDPD1, YPEL2, LINC01476*
			59,336,282	59,545,765	DupInv	0.21	C	*YPEL2, LINC01476*
**US-SV10**	DupINVdup	17	59,220,112	59,288,295	Dup	0.07	B	*GDPD1*
			59,220,112	59,478,159	Inv	0.26	B,C,D	*GDPD1, YPEL2, LINC01476*
			59,362,562	59,478,159	Dup	0.12	D	*YPEL2, LINC01476*

SV, Structural variant; Type, Complex structural rearrangements specified as duplications (dup), inversions (inv) or complex events; Start, End, Genomic positions of structural rearrangements according to GRCh38/hg38; Size, Size of structural rearrangement in Mb; Genomic regions, Genomic regions annotated as illustrated in **Figure 3**.

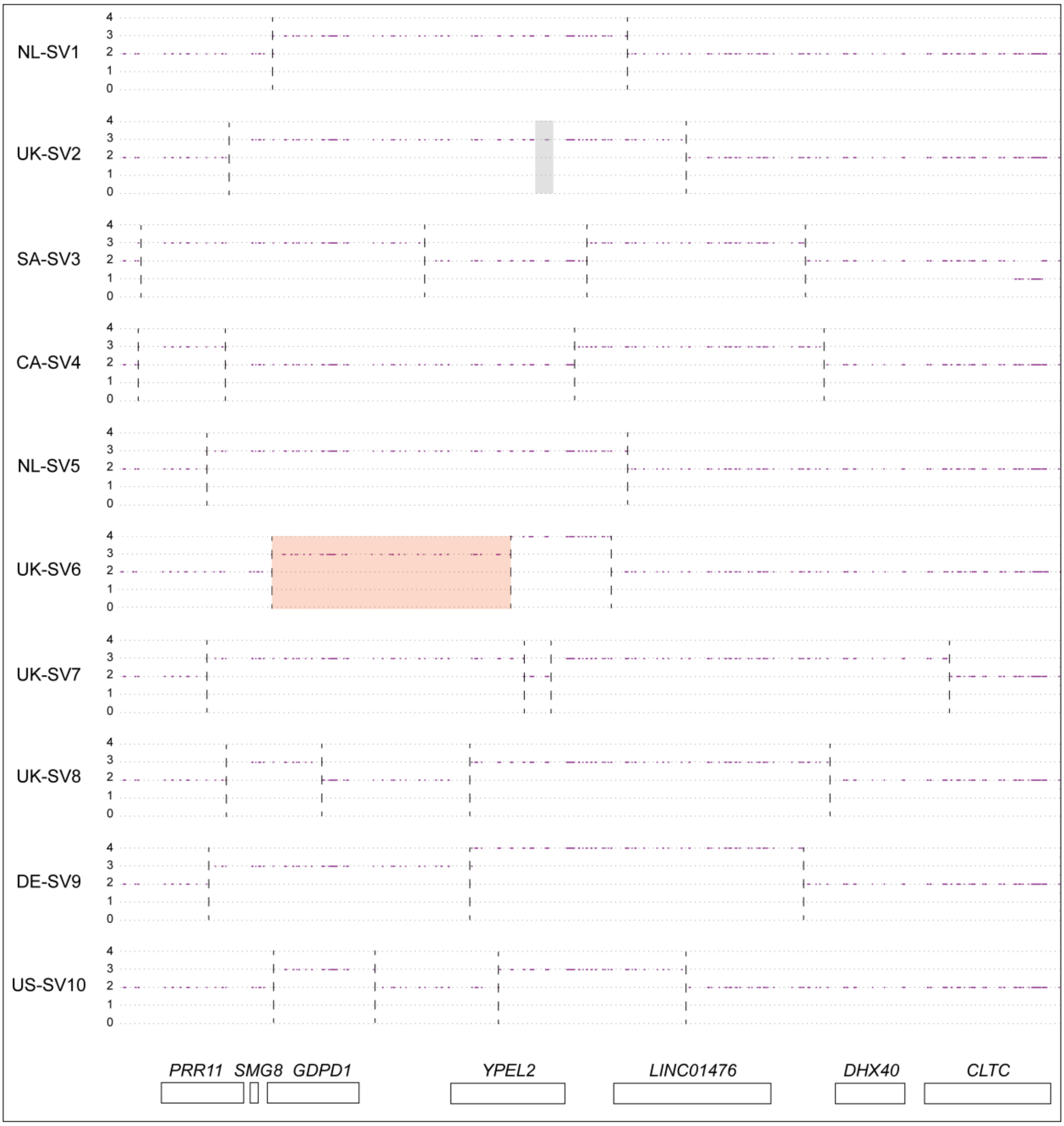

Supplementary Figure 1: Overview of the SNP array data from all currently known RP17-SVs. The eight previously described RP17-SVs (NL-SV1 – UK-SV8) and the two novel RP17-SVs (DE-SV9 and US-SV10) described in this study show differences in the copy number of different genes within the RP17 locus and unique SV breakpoints. The dashed lines represent the SV breakpoints based on the SNP array data. For UK-SV2, two additional breakpoints were expected in *YPEL2*, but this small copy number change was not detected in the SNP array data (indicated by the grey box). For UK-SV6, SNP-array results suggested an incomplete triplication (indicated by the red box) of the genomic segment and an additional mutational breakpoint was suggested. The figure was generated using the copy number state data type from the ChAS software.

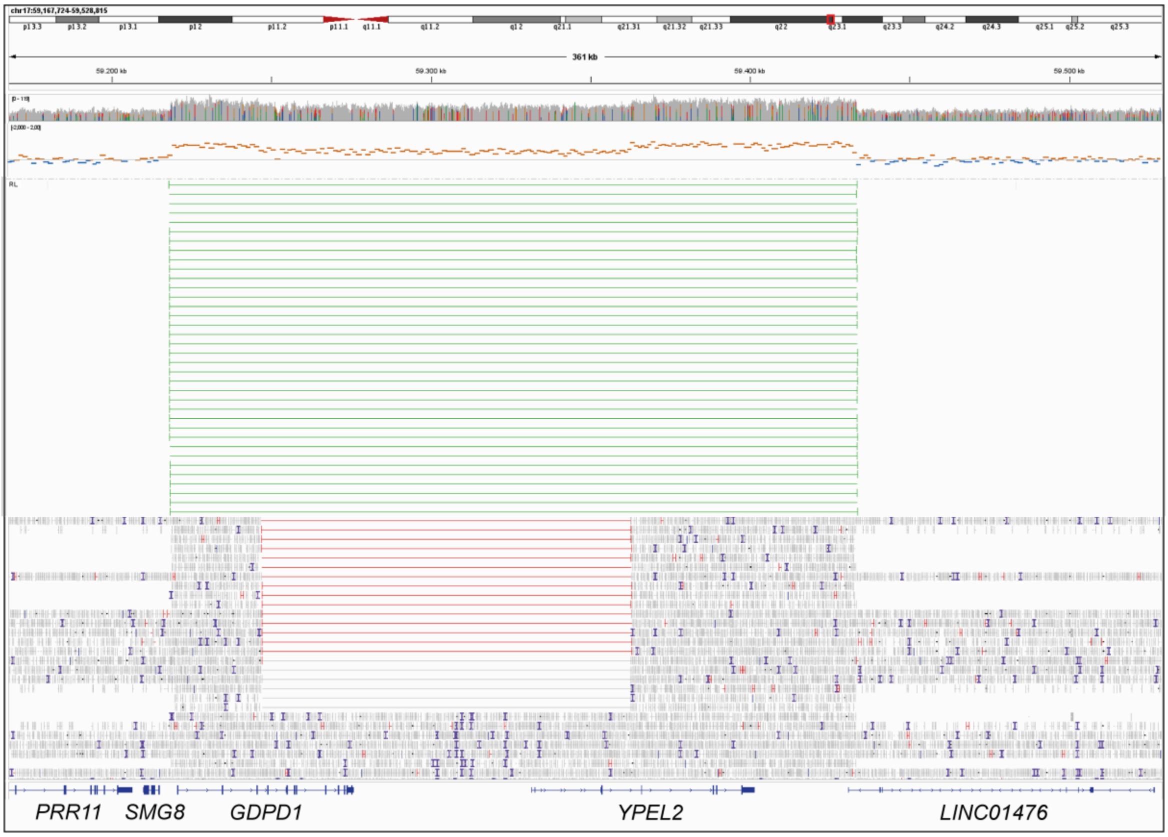

Supplementary Figure 2: Reanalysis of genome sequencing data UK-SV6 confirms duplication-triplication event. Interrogation of the genome sequencing data of UK-SV6 in the Integrative Genomics Viewer (IGV) revealed changes in coverage depth (top panel) and split reads (green (tandem duplication) and red reads (deletion)) corresponding to the two mutation-specific breakpoints predicted by SNP-array. One breakpoint corresponds with the previously reported triplication breakpoint (previously coined “B1”). Based on SV- and CNV-calls a duplication-triplication event could be predicted. The presence of both breakpoints was confirmed by breakpoint-specific PCR and validated using Sanger sequencing.

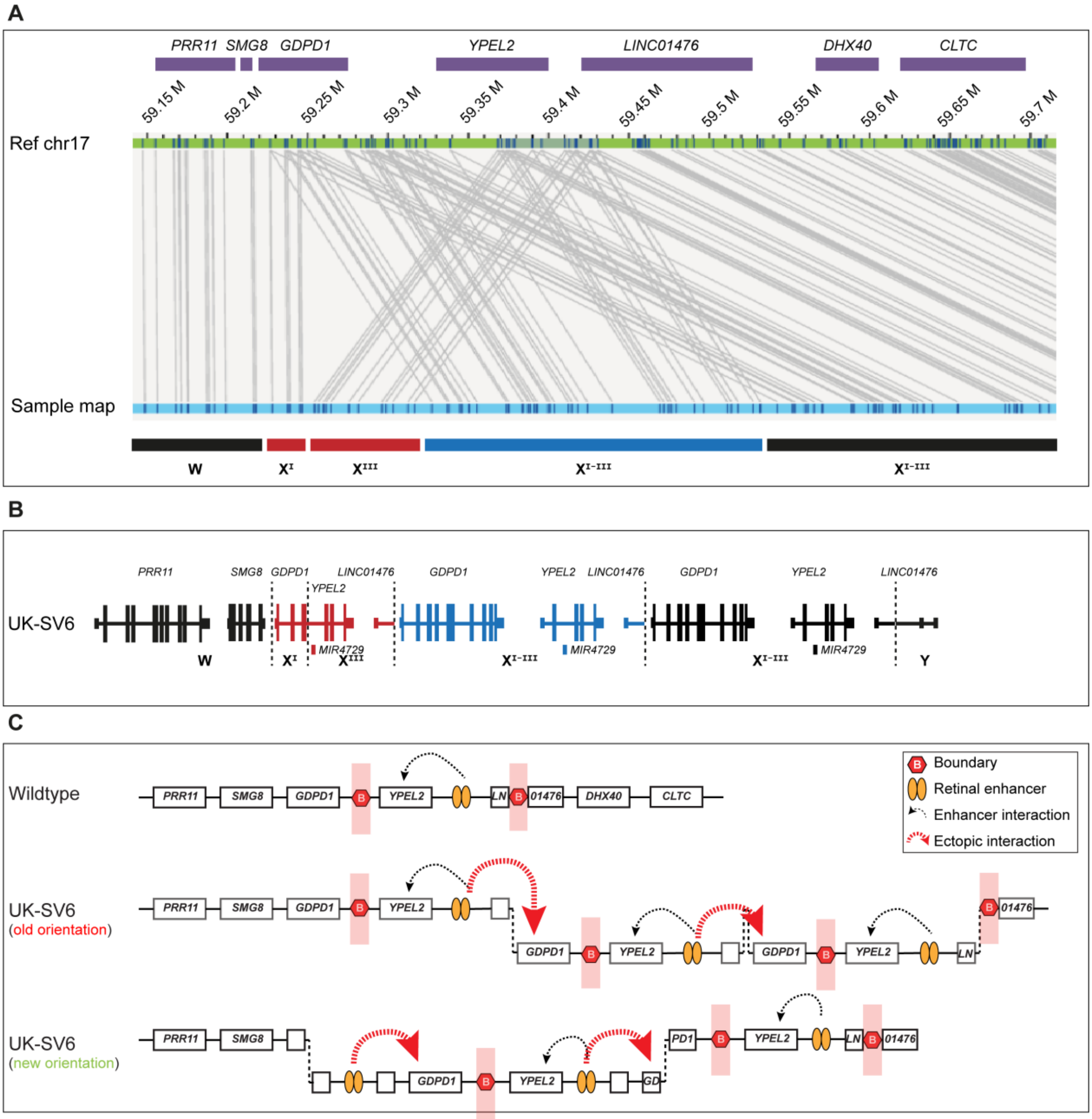

Supplementary Figure 3: Optical genome mapping resolves structure and orientation of UK-SV6. Optical genome mapping (OGM) was performed for an affected individual harboring UK-SV6 to completely resolve the updated structure of UK-SV (i.e., which presumed triplicated segment (segment "X") harbors a deletion?). (A) The sample genome map is mapped against the ref chr17, revealing the triplicated segments ("X-I" and "X-III") spanning a deleted fragment, and the duplicated segment ("X-II"), the mutational breakpoints (B1 and B2) and the order of the segments. (B) Updated nomenclature and schematic representation of UK-SV6. The blue parts represent duplicated or triplicated segments, the red parts span a deletion event. (C) Schematic modeling of the revised UK-SV6. Based on the original description of UK-SV6 in our earlier publication (1), we predicted that two copies of *GDPD1* could enter active neo-TAD domains as a consequence of the triplication event (UK-SV6, old orientation). Based on the revised duplication-triplication structure of UK-SV6, we predict that the same ectopic contacts are still possible between the two copies of *GDPD1* and the retinal enhancer elements and that these contacts are not affected by the additional deletion event (UK-SV6, new orientation). The SV is classified as (likely) pathogenic. The red arrow indicates the predicted creation of ectopic contacts between the retinal enhancer elements and *GDPD1* promoter.

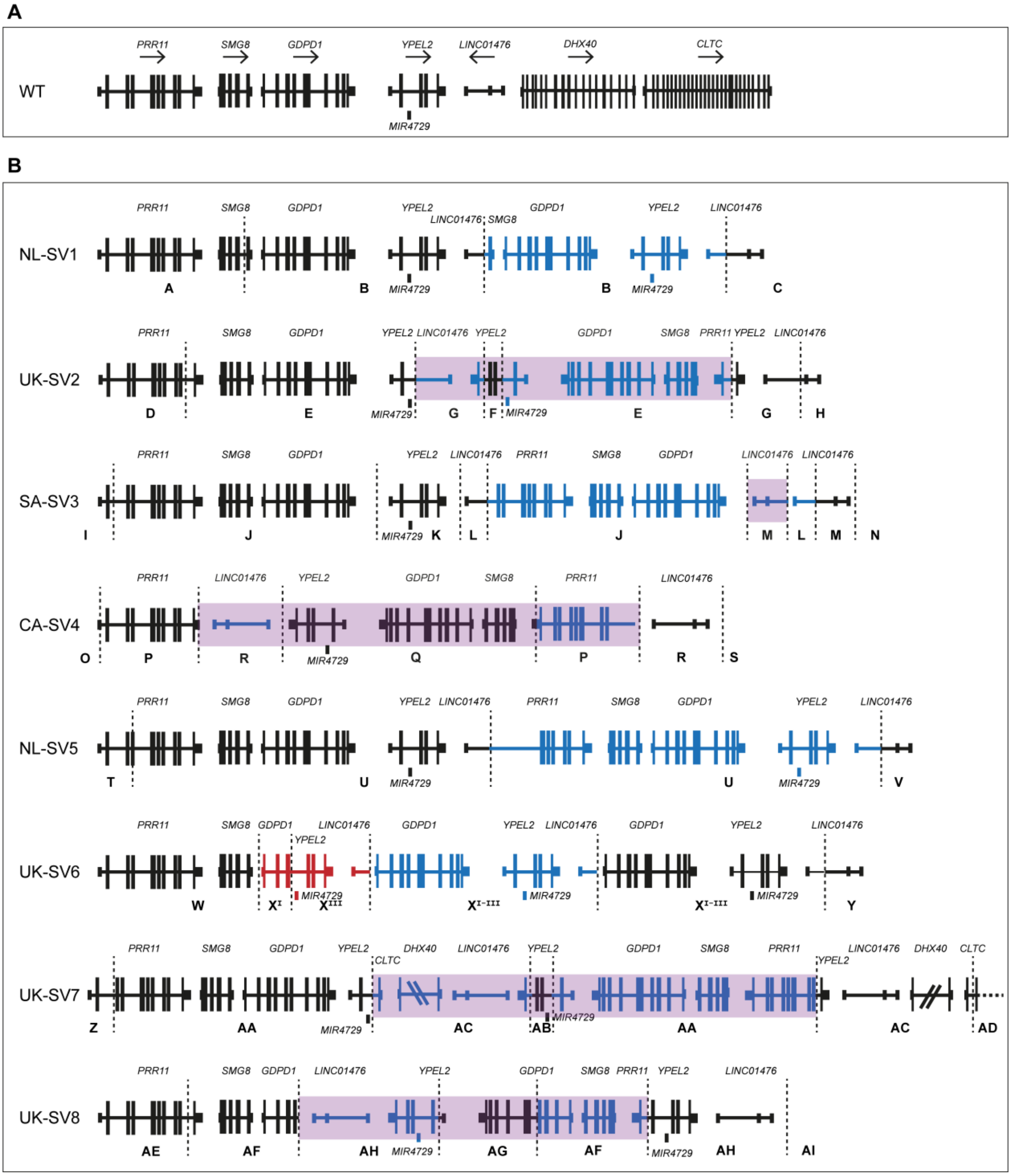

Supplementary Figure 4: Overview of previously reported pathogenic structural variants in the RP17 locus. (A) Schematic representation of wildtype RP17 locus. (B) Schematic overview of eight known RP17 structural variants (SV1-SV8) as previously published (1). Based on recent data acquired using our approach, (**Supplementary Results**), the structure and nomenclature of UK-SV6 has been revised compared to the previous report (1). Breakpoints are indicated with dashed lines. Blue segments represent duplicated or triplicated regions, red segments span an internal deletion, whereas inversions are highlighted in purple. The size of *DHX40* is reduced and *CLTC* is partially shown for the purpose of this figure. Figure adapted from de Bruijn et al. 2020 (1).

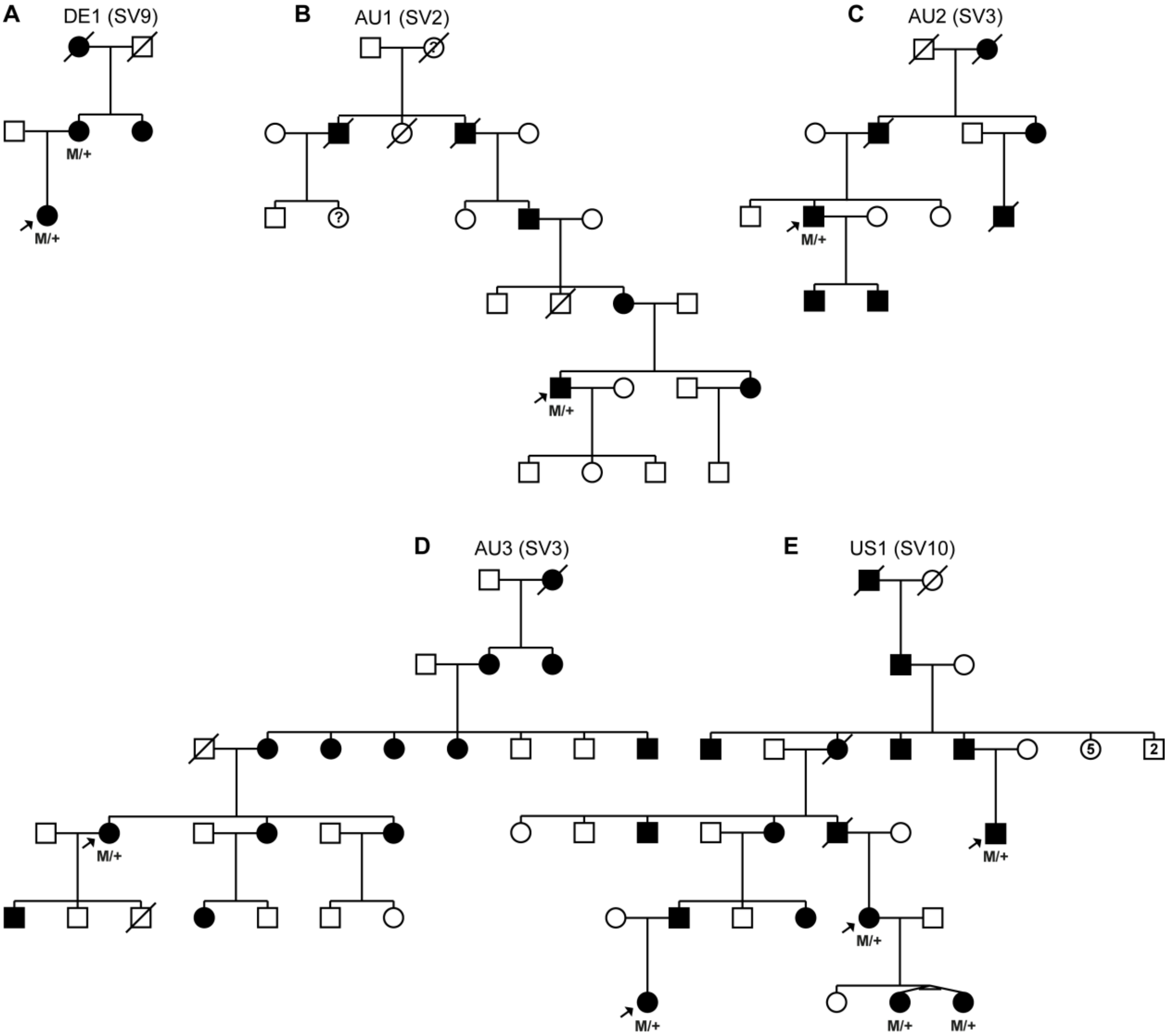

Supplementary Figure 5: Autosomal dominant retinitis pigmentosa (adRP) families with novel or previously reported structural variants within the RP17 locus. Index cases are indicated with an arrow. Individuals in which the presence of the structural variant was confirmed with mutation-specific breakpoint PCR are indicated with "M/+". ?, phenotype unknown. DE-SV9 was identified in the German adRP family DE1, UK-SV2 was identified in Australian adRP family AU1, SA-SV3 was identified in Australian adRP families AU2 and AU3, and US-SV10 was identified in adRP family US1 from the United States. For families AU1, AU2 and AU3, segregation analysis of SA-SV3 could not be performed due to the unavailability of DNA from affected family members. However, SA-SV3 has previously been reported as pathogenic in four large South-African families, where it was confirmed to cosegregate with the adRP phenotype (1).

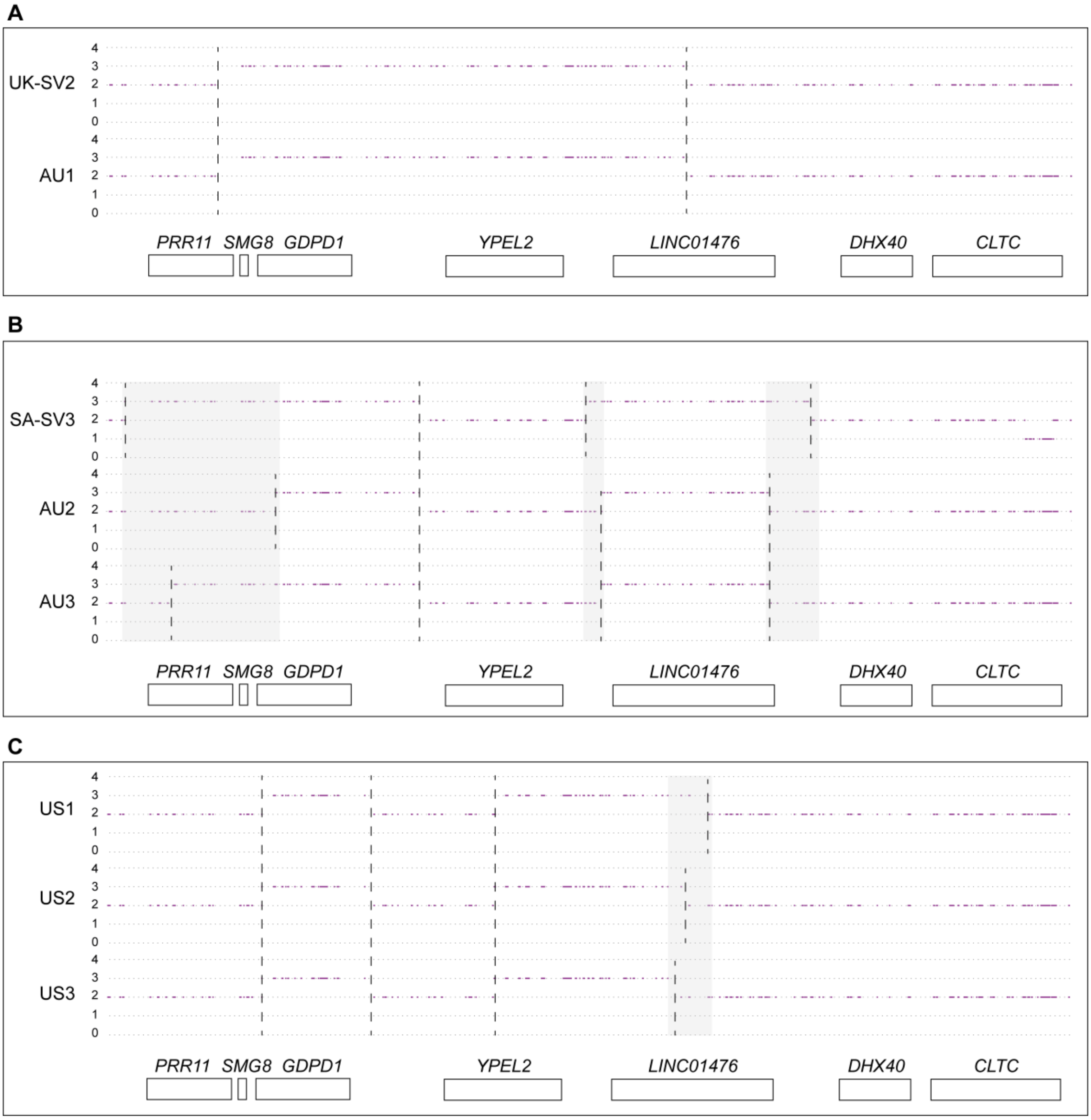

Supplementary Figure 6: Comparison to reference SNP array data reveals known and novel RP17-SVs in six probands. The dashed lines represent the SV breakpoints based on the SNP array data. (A) The SNP array plot from a proband from Australia (AU1) matched the reference plot for UK-SV2. (B) The SNP array plots for two probands from Australia (AU2 and AU3) were similar to the reference plot for SA-SV3. There were observed differences in three breakpoints (indicated by the grey boxes) by this method, but SA-SV3 was confirmed as the SV in these two probands with a mutation-specific breakpoint PCR. (C) The SNP array plots from three probands from the United States (US1-3) did not resemble previous SNP array data, but showed an almost identical pattern and the probands were found to carry a novel SV (US-SV10). The figure was generated using the copy number state data type from the ChAS software.

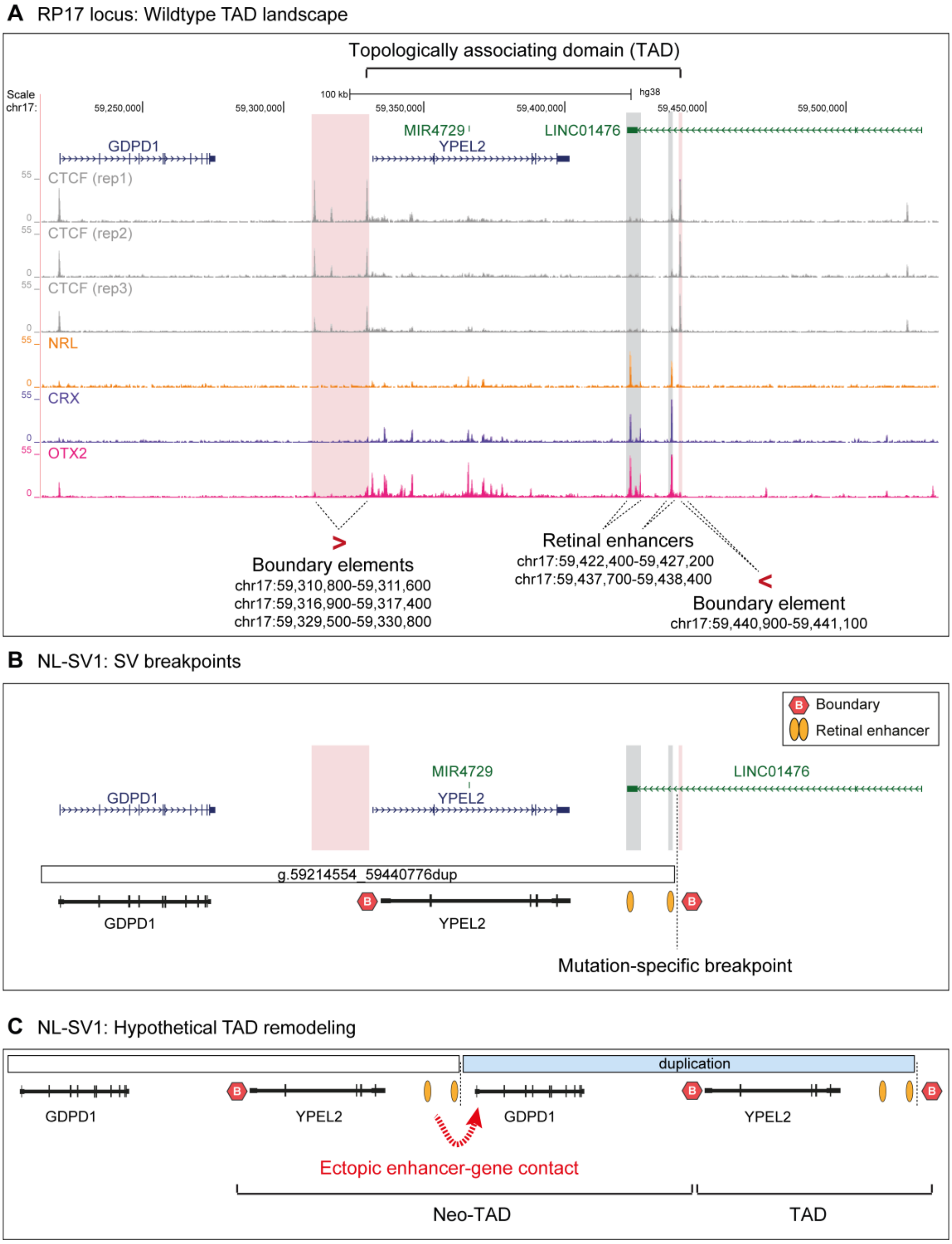

Supplementary Figure 7: 3D chromatin organization of RP17 locus. (A) Overview of topologically associating domain (TAD) CTCF-boundaries and orientation, gene positions and retinal-specific enhancers that are present within the RP17 locus based on publicly available epigenetic datasets (4). The CTCF ChIP-seq human retina data indicate that *YPEL2* is located within an insulated TAD, with several CTCF-binding sites present on the 5'side of the *YPEL2* and a single CTCF-binding site on the 3'side of the TAD domain. Red arrows are used to indicate the CTCF-boundary orientations. (B) Mapping of the breakpoints of NL-SV1 within the RP17 locus. (C) Hypothetical consequences and rearrangement of TAD structures caused by NL-SV1. NL-SV1 creates the formation of a neo-TAD structure including ectopic contacts between retinal active enhancers and the *GDPD1* promoter. Rep, biological replicate.

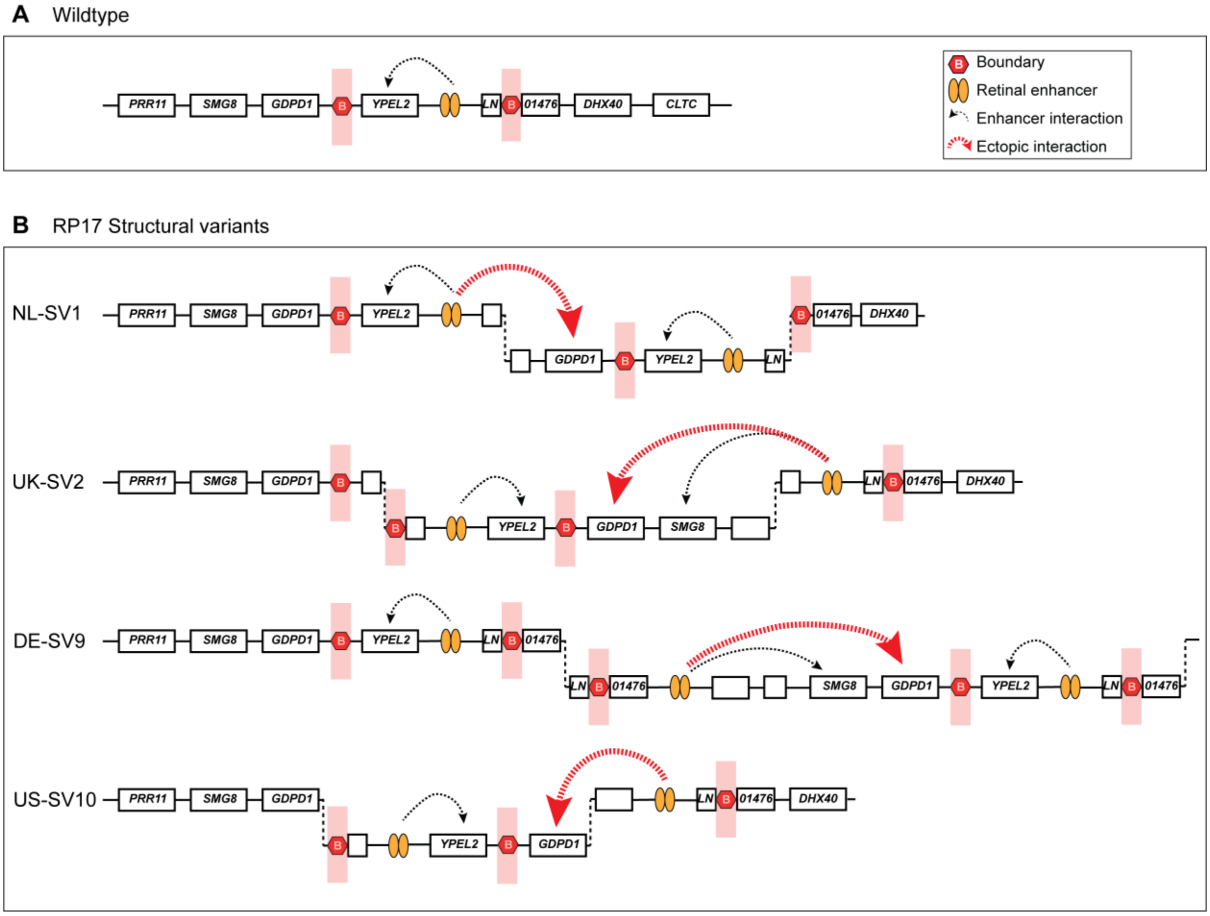

Supplementary Figure 8: RP17-SVs are predicted to create new TAD-domains with ectopic contacts between retinal-specific enhancers and *GDPD1*. Schematic modeling of the RP17 locus is based on publicly available retina-specific epigenetic datasets and is performed as previously described (1). NL-SV1 and UK-SV2 are included as examples. The red arrow indicates the predicted creation of ectopic contacts between the retinal enhancer elements and *GDPD1* promoter.

SUPPLEMENTARY REFERENCES

1. de Bruijn SE, Fiorentino A, Ottaviani D, Fanucchi S, Melo US, Corral-Serrano JC, et al. Structural Variants Create New Topological-Associated Domains and Ectopic Retinal Enhancer-Gene Contact in Dominant Retinitis Pigmentosa. Am J Hum Genet. 2020;107(5):802-14.
2. Robinson JT, Thorvaldsdóttir H, Winckler W, Guttman M, Lander ES, Getz G, Mesirov JP. Integrative genomics viewer. Nat Biotechnol. 2011;29(1):24-6.
3. de Bruijn SE, Rodenburg K, Corominas J, Ben-Yosef T, Reurink J, Kremer H, et al. Optical genome mapping and revisiting short-read genome sequencing data reveal previously overlooked structural variants disrupting retinal disease−associated genes. Genetics in Medicine. 2023;25(3):100345.
4. Cherry TJ, Yang MG, Harmin DA, Tao P, Timms AE, Bauwens M, et al. Mapping the cis-regulatory architecture of the human retina reveals noncoding genetic variation in disease. Proc Natl Acad Sci U S A. 2020;117(16):9001-12.
